# Identification of a key role of widespread epigenetic drift in Barrett’s esophagus and esophageal adenocarcinoma

**DOI:** 10.1186/s13148-017-0409-4

**Published:** 2017-10-16

**Authors:** E. Georg Luebeck, Kit Curtius, William D. Hazelton, Sean Maden, Ming Yu, Prashanthi N. Thota, Deepa T. Patil, Amitabh Chak, Joseph E. Willis, William M. Grady

**Affiliations:** 1 Program in Computational Biology, Fred Hutchinson Cancer Research Center, Seattle, WA 98109 USA; 2 Centre for Tumour Biology, Barts Cancer Institute, Queen Mary University of London, Charterhouse Square, London, EC1M 6BQ UK; 3 Clinical Research Division, Fred Hutchinson Cancer Research Center, Seattle, WA 98109 USA; 4 Department of Gastroenterology, Digestive Disease & Surgery Institute, Cleveland Clinic, Cleveland, OH 44195 USA; 5 Department of Pathology, Cleveland Clinic, Cleveland, OH 44195 USA; 6 University Hospitals Case Medical Center, Case Western Reserve University School of Medicine, Cleveland, OH 44106 USA; 7 Department of Medicine, University of Washington School of Medicine, Seattle, WA 98195 USA

**Keywords:** Barrett’s esophagus (BE), Esophageal adenocarcinoma (EAC), Tissue age, Epigenetic drift, DNA methylation, Neoplastic progression, Transcriptional repression in cancer, Endogenous retroviruses (ERVs)

## Abstract

**Background:**

Recent studies have identified age-related changes in DNA methylation patterns in normal and cancer tissues in a process that is called epigenetic drift. However, the evolving patterns, functional consequences, and dynamics of epigenetic drift during carcinogenesis remain largely unexplored. Here we analyze the evolution of epigenetic drift patterns during progression from normal squamous esophagus tissue to Barrett’s esophagus (BE) to esophageal adenocarcinoma (EAC) using 173 tissue samples from 100 (nonfamilial) BE patients, along with publically available datasets including The Cancer Genome Atlas (TCGA).

**Results:**

Our analysis reveals extensive methylomic drift between normal squamous esophagus and BE tissues in nonprogressed BE patients, with differential drift affecting 4024 (24%) of 16,984 normally hypomethylated cytosine-guanine dinucleotides (CpGs) occurring in CpG islands. The majority (63%) of islands that include drift CpGs are associated with gene promoter regions. Island CpGs that drift have stronger pairwise correlations than static islands, reflecting collective drift consistent with processive DNA methylation maintenance. Individual BE tissues are extremely heterogeneous in their distribution of methylomic drift and encompass unimodal low-drift to bimodal high-drift patterns, reflective of differences in BE tissue age. Further analysis of longitudinally collected biopsy samples from 20 BE patients confirm the time-dependent evolution of these drift patterns. Drift patterns in EAC are similar to those in BE, but frequently exhibit enhanced bimodality and advanced mode drift. To better understand the observed drift patterns, we developed a multicellular stochastic model at the CpG island level. Importantly, we find that nonlinear feedback in the model between mean island methylation and CpG methylation rates is able to explain the widely heterogeneous collective drift patterns. Using matched gene expression and DNA methylation data in EAC from TCGA and other publically available data, we also find that advanced methylomic drift is correlated with significant transcriptional repression of ~ 200 genes in important regulatory and developmental pathways, including several checkpoint and tumor suppressor-like genes.

**Conclusions:**

Taken together, our findings suggest that epigenetic drift evolution acts to significantly reduce the expression of developmental genes that may alter tissue characteristics and improve functional adaptation during BE to EAC progression.

**Electronic supplementary material:**

The online version of this article (10.1186/s13148-017-0409-4) contains supplementary material, which is available to authorized users.

## Background

Research into the connection between aging and cancer is being fueled by advances in molecular profiling of age-related processes such as chronic inflammation, accumulation of somatic DNA/mtDNA mutations, and epigenetic changes in tissues in which cancers arise [1]. Two distinct, albeit not entirely independent, concepts have emerged recently that relate changes in DNA methylation to biological tissue age. The first is based on the discovery (of sets) of CpG dinucleotides (CpGs) in the genome that are subject to age-dependent, possibly complex changes in methylation levels that, in aggregate, correlate strongly with chronological age [2–4]. We refer to these types of CpGs as *clock-CpGs.* A second and simpler concept is based on the observation of gradual age-related changes in methylation levels at specific CpG sites or CpG-rich regions, a process commonly referred to as *epigenetic* or *methylomic drift* [5–11]. For example, some CpG islands show very low methylation levels early in life but are known to become gradually methylated over time as a result of sporadic de novo methylation events during DNA replication. We identify these as *drift CpGs*. It is worth pointing out that data supporting these concepts come mainly from cross-sectional studies that include individuals of different age. In contrast, individual-level (longitudinal) drift, unless studied directly in select individuals over time as we have collected for this study, is typically inferred from population drift.

In a recent study, we used a combination of cross-sectional and longitudinally collected biopsy samples to identify a set of highly correlated CpGs in premalignant Barrett’s esophagus (BE) tissue that undergo differential epigenetic drift relative to normal squamous (NS) tissue [12]. This study arrived at a set of 67 drift CpGs that show significant age-related methylation differences between NS and BE and was used as an epigenetic clock to estimate the time of BE onset. Unlike previous epigenetic clocks that were constructed to predict the age of an individual, the BE tissue-specific clock model was designed to infer unknown tissue ages. Because BE is essentially asymptomatic, it is usually not known how long a patient has lived with BE. However, the time a patient has lived with BE may be considered a risk factor since older BE tissue has had more time for cancer to evolve compared to younger BE tissue which is more likely to be free of neoplastic changes [13, 14]. Although the earlier study was the first to develop an epigenetic clock for BE tissue age, we did not evaluate the full scope of epigenetic drift occurring in BE following its formation, nor its dynamics or functional consequences at the genomic level.

The aims of this study are to characterize individual heterogeneity and genome-wide patterns of age-related epigenetic drift in tissue samples from BE and EAC patients, to develop a mechanistic understanding of methylation dynamics during tissue aging, including the degree of cooperativity and possible presence of nonlinear feedback within CpG islands during the temporal evolution of epigenetic drift, and to explore the impact of advanced drift on gene expression in EACs for which we have both gene expression and methylation data.

To accomplish the aims of this study, it was necessary to analyze methylation drift patterns from an extensive collection of NS, BE, and EAC biopsy tissue samples, including 173 tissue samples from 100 nonprogressed and progressed (nonfamilial) BE patients, along with methylation and gene expression data in 87 EAC from The Cancer Genome Atlas (TCGA) [15] and in 47 EAC + 4 BE samples previously analyzed by Krause et al. [16].

## Results

Using data from the HM450 methylation arrays (see “Methods”), we aimed to better characterize the full extent of epigenetic drift occurring in BE and the temporal dynamics of epigenetic drift and to explore the impact of advanced drift on gene expression in EAC for which we have both gene expression and methylation data. Methylation levels are measured either in terms of a *β* value (methylation fraction) or *M* value (logit_2_(β)) as indicated in the text.

### Genomic scope of drift

**Fig. 1:**
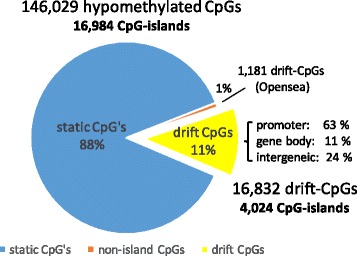
Proportion of CpGs and CpG islands that drift differentially in Barrett’s esophagus vs. normal squamous (NS) esophagus among over 146 k hypomethylated probes in NS tissue

Out of 146,029 hypomethylated CpG probes in normal squamous (NS) tissue, we identified 18,013 (12%) probes that have significant positive correlation and 560 (0.4%) that have a significant negative correlation (*q* < 0.01) with the mean differential drift (relative to NS levels) of 67 previously validated drift CpGs in 64 BE samples from patients without a diagnosis of dysplasia or cancer (see “Methods”). In contrast, out of 133,857 CpG probes that were hypermethylated in NS tissue, only 795 (0.6%) probes correlated positively and 3402 (2.5%) probes correlated negatively with the mean methylation drift levels of our 67 probe reference clock (Fig. 1). Thus, significant differential drift in BE involves thousands of CpGs, occurring predominantly in hypomethylated regions that are associated with CpG islands, affecting 4024 (24%) of the 16,984 hypomethylated CpG islands in NS tissue. In contrast, we found only 7% of the identified drift CpG probes to be “open sea,” i.e., isolated in the genome [17], compared to about 10% in the hypomethylated normal background.

The majority (63%) of islands that include drift CpGs are associated with gene promoter regions, i.e., they involve a transcription start site (TSS200 or TSS1500), while only 11% of islands that undergo drift overlap with the gene body, compared to 73% (TSS-associated) and 10% (body-associated) CpG islands on the HM450 array, respectively. In contrast, the relative abundance of intergenic CpG islands is significantly higher among CpG islands that undergo drift compared to the fraction of intergenic CpG islands found on the array (24 vs. 16%, see Fig. 1).

**Fig. 2:**
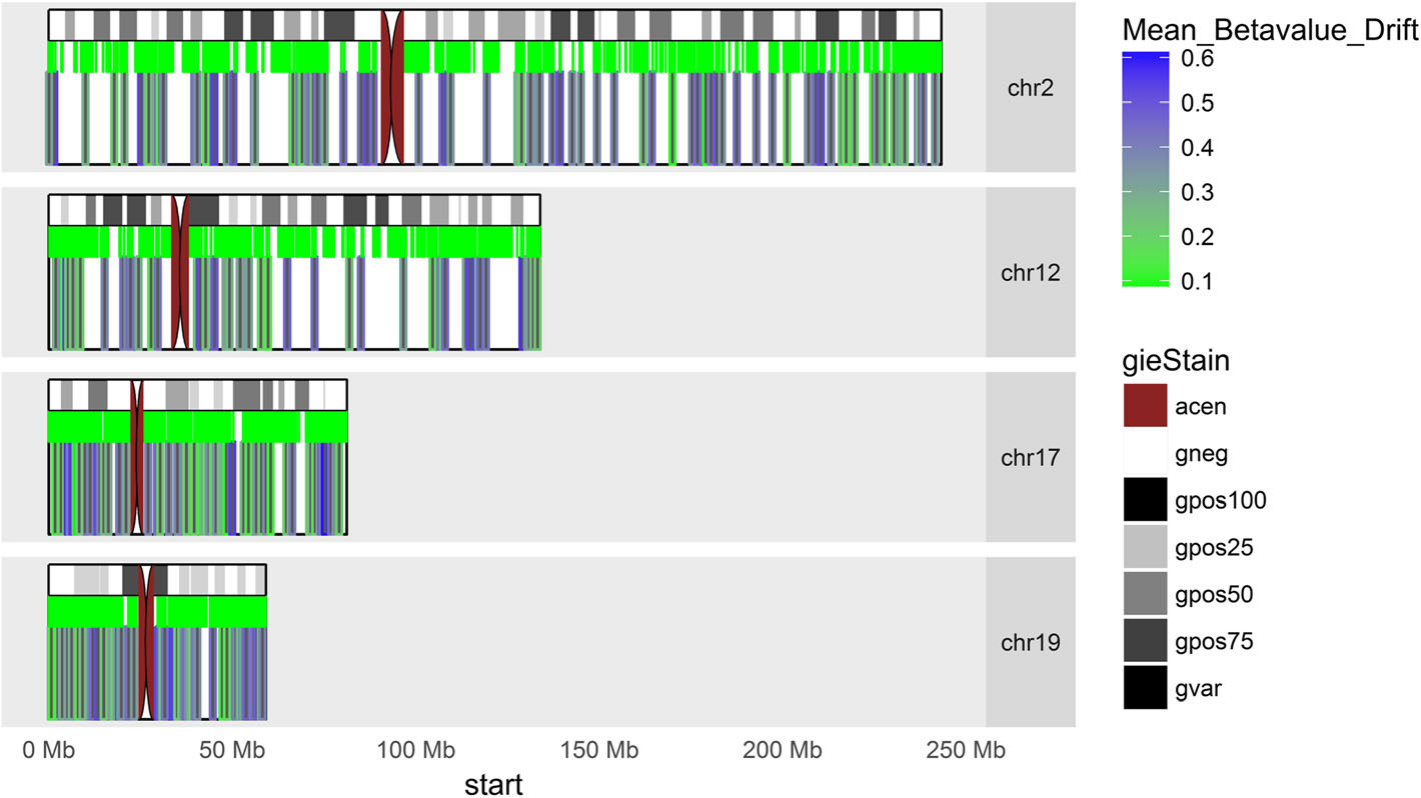
Representative karyographs of four autosomes. Chromosomes 2 and 12 exhibit typical methylomic drift patterns while chromosomes 17 and 19 exhibit high-density methylomic drift. Top track: chromosome banding. Middle track: array-based CpG island positions. Bottom track: positions of CpG islands that undergo methylomic drift in 64 BE samples (mean levels color-coded)

**Fig. 3:**
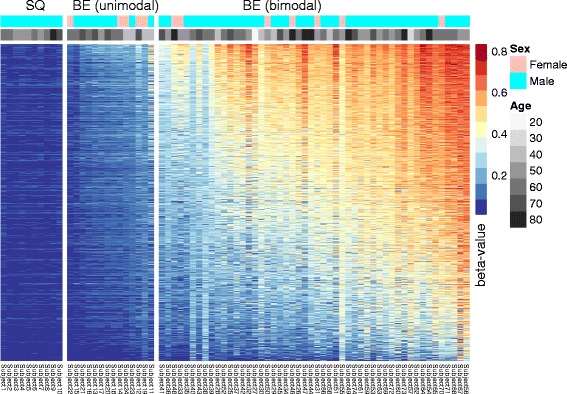
CpG island-level methylation heatmap (*β* values) of 1317 drift CpG islands (rows) and 10 NS and 64 non-dysplastic BE samples (columns) ordered by their respective means. See text for details

Out of 16,832 island-based (BE-specific) drift CpGs we identified, 1317 unique CpG islands with 5 or more drift CpGs per island (comprising a total of 11,425 drift CpGs). Figure 2 presents a karyograph of 4 autosomes indicating the genomic locations and mean *β* values for these islands across the 64 BE samples (Additional file 1: Figure S4 for all 22 autosomes). Figure 3 shows a heatmap of the island-level mean *β* values of the 1317 drift-associated CpG islands for the first 10 NS tissue samples, and all 64 BE samples used to identify CpG probes undergoing drift. For this map, both CpG islands and tissue samples were ordered by their respective mean values. As expected, all 10 normal control tissue samples show no island-level drift. In contrast, we see significant heterogeneity in mean methylation levels of these CpG islands ranging from < 20 to > 80% methylation across the 64 BE samples. With the notable exception of a group of samples that have undergone minimal drift, most BE samples show bimodal patterns of drift where some islands appear to linger at low levels and others show advanced drift. We later use the following categorization for the observed drift patterns in BE and EAC: unimodal low drift (group L), bimodal high drift with a major mode *β* > 50% (group H) and the remaining bimodal intermediate drift (group I).

### Pairwise correlations between island-associated CpGs

**Fig. 4:**
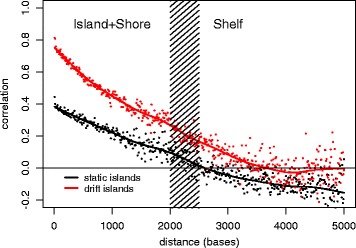
Pairwise correlations between island-CpGs and other CpGs designated as island, shore, and shelf, associated with the same island, as a function of genomic distance at a resolution of 10 bp. “Static” (nondrifting) CpG islands (black), drift-associated islands (red). Shaded area represents the approximate boundary location between shores and shelves

CpG islands are considered functional genomic units that may exert transcriptional control by their collective state of methylation rather than through individual CpG sites. To demonstrate this collective behavior in an island-level DNA methylation, we evaluated the pairwise correlations between all island CpGs that are hypomethylated in NS tissue. In general, for static islands (that do not show significant drift), pairwise correlations are moderate (< 0.5) across the span of an island and exhibit anti-correlations near and beyond the island boundaries (Fig. 4). In contrast, island CpGs that drift have stronger pairwise correlations reflecting a collective response of these CpGs to drift consistent with processive DNA methylation maintenance [18, 19].

Figure 4 also shows that the pairwise correlations decay with genomic distance and, for drift CpGs, extend further into the island shelves than static CpGs. Islands that show tissue age-related drift are also significantly larger (in terms of genomic length) than static islands. The mean sizes of the static vs. drift CpG islands were 0.9 vs 1.1 kb, respectively (*p* = 2 × 10^−10^, two-sided *t* test).

### Bimodal nature of epigenetic drift in BE and EAC

**Fig. 5:**
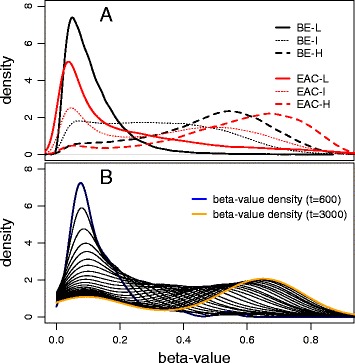
**a** Typical drift patterns for BE and EAC samples by type of *β* value distribution. Shown are the methylation distributions for 11,425 island-associated drift CpGs (minimum of 5 drift CpGs per island). The three drift groups are based on unimodal low drift (group L), bimodal intermediate drift (group I), and bimodal high (group H). **b** Simulated methylation densities (arbitrary time scale) for an island-like region of 50 CpGs and 1000 cells mimicking the array-based measurements of epigenetic drift in panel **a**. Details provided in text

To see whether methylomic drift is uniformly distributed within our BE and EAC samples, we examined *β* value distributions for a subset of island-associated drift CpGs with a minimum of 5 detected drift CpGs per island (11,425 drift CpGs in total) for 64 BE and 24 EAC samples from the BETRNet (see “Methods”). Consistent with the patterns seen in Fig. 3, these individual-level distributions show signatures that fall into the arbitrary three types: with unimodal distributions showing low or no drift (group L), distinctly bimodal distributions with intermediate drift (both modes *β* < 0.5, group I), or bimodal with a major mode near or above *β* = 0.5 and a minor mode at lower levels (group H). (See Fig. 5a, for an aggregated view of the samples in these groups). While the distributions are similar for BE and EAC, EAC show more advanced drift in the third group (bimodal high) which may be attributed to EAC patients being on average older than the BE patients (68 vs 62 years, respectively), or to the fact that EAC undergoes more frequent stem cell divisions thereby increasing replication-coupled de novo methylation, or to the possibility that BE arises earlier in patients with EAC compared to patients who have not progressed to dysplasia or EAC. We found similar unimodal/bimodal drift signatures in 87 EAC from TCGA and in a combined set of 19 BE and 47 EAC tissue samples provided by Krause et al. [16] (GEO accession number: GSE72874).

### Advanced drift is associated with low tumor stage

Using tumor stage information from the TCGA, we found a statistically significant association (*p* value = 0.024; Fisher’s exact test) of low tumor stage (AJCC stage I) vs advanced stage (AJCC stage III and higher) with the type of drift pattern (group H vs group L + I). Specifically, low-stage tumors are more prevalent in group H compared with group L + I among the 74 TCGA EAC for which tumor stage information was available (odds ratio 6.0 (1.1–63.3)).

### Differential gene expression by drift group

To see whether gene expression patterns (at the mRNA level) differed between EAC samples that showed minor (unimodal low) drift and samples that showed advanced methylomic drift on a gene by gene basis, we matched 1240 drift CpG islands (out of 1317 CpG islands with 5 or more drift CpGs per island) with one or more (overlapping) genes to evaluate the relationship between gene expression and island-level methylation for the TCGA and the Krause et al. data sets. Specifically, we identified differentially expressed genes for which expression differed significantly between low- and high-drift samples by setting a threshold of *β* = 0.2 to delineate the two groups and using a two-sided Mann-Whitney-Wilcoxon test (*q* < 0.01) on normalized gene expression data.

In total, we identified 200 genes that were significantly underexpressed in the advanced drift group while only 10 genes were significantly overexpressed (Additional file 2: Table S1). Independently, we found 35 genes that were significantly repressed and none that were significantly overexpressed among 51 (47 EAC + 4 BE) samples provided by Krause et al. [16]. Importantly, several genes (20/35) that were found repressed in the smaller study by Krause et al. were also found repressed in TCGA (Additional file 2: Table S1). In particular, the gene most significantly repressed in TCGA (*q* = 5 × 10^−9^) was also ranked most significantly repressed in the data provided by Krause et al., *CHFR* (checkpoint with forkhead and ring finger domains), a mitotic stress checkpoint gene with tumor suppressive function that has been identified in a wide range of cancers [20, 21], and most recently as a significantly silenced gene in a large clustering analysis of esophageal adenocarcinoma [22]. This striking asymmetry between gene expression changes and methylomic drift is consistent with parallel findings that CpG promoter hypermethylation in cancers often is correlated with gene-silencing [6]. A Gene Ontology (GO)-based over-representation analysis using the Database for Annotation, Visualization and Integrated Discovery (DAVID) shows a highly significant greater than threefold enrichment of sequence-specific DNA binding transcription factor activity (*p* = 2 × 10^−9^, Additional file 2: Table S2). The most prominent group identified by this analysis is a family of repressive Krueppel-associated box (*KRAB*) domain zinc finger (ZNF) transcription factors (greater than sixfold, *p* = 1.3 × 10^−15^, Additional file 2: Table S2). *KRAB*-mediated transcriptional repression involves the binding of the *KRAB* domain to co-repressors potentially resulting in heterochromatin formation and silencing of endogenous retroviruses [23, 24].

### Evidence for a threshold effect leading to bimodal drift

**Fig. 6:**
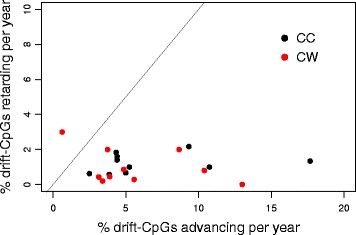
CpG methylation transition rates based on paired longitudinal biopsies (collected at least 3–4 years apart) from the Cleveland Clinic (CC) (black) and Case Western (CW) (red). Forward (increasing) methylation transition rates represent the annual rate of CpG probes advancing past a threshold of *β* = 0.2, while retarding transition rates represent the fraction of CpG probes transitioning from high to low methylation (below *β* = 0.2)

The drift patterns shown in Fig. 5a for BE and EAC samples suggest nonlinear drift dynamics in BE tissues. Specifically, the presence of a persistent mode in the drift distribution at low levels (*β* < 0.2) is indicative of a threshold below which drift is suppressed but advances rapidly once the mean level is surmounted. To validate that epigenetic drift occurs in our longitudinal samples (20 patients with 2 biopsies each separated by at least 3–4 years), we determined for each individual at two time points the number of drift CpGs that remained below (n_11_), respectively, the number that remained above (n_22_), and the number of drift CpGs that had crossed the threshold from low to high at *β* = 0.2 (n_12_) and, vice versa, from high to low (n_21_). The results, including the % fraction of drift CpGs advancing, n_12_/(n_12_ + n_11_), and the % fraction retarding between the two time points, n_21_/(n_21_ + n_22_), are listed in Additional file 2: Table S3 and shown as annual rates in Fig. 6. For 19/20 patients, we detect greater methylation flow from sub-threshold levels to higher levels at the second (later) biopsy compared to flow in the opposite direction.

We note that these findings are surprisingly consistent with the unimodal-to-bimodal epigenetic drift predictions made by Sontag et al. [8] who proposed a mathematical model that included a nonlinear relationship between de novo methylation and the ambient level of methylation present in a region of CpGs. To demonstrate that such a model results in unimodal-to-bimodal drift transitions over time, we explicitly simulated sporadic de novo methylation on an island of 50–100 CpGs, independently in 1000 cells, mimicking crudely the cell population in the tissue samples. Each CpG was assumed to be in a binary state (0/1 of being (un)methylated), and the states of the CpGs initially (at time *t* = 0) were sampled from a binomial distribution with probability 0.06 which equals the mean methylation level in our NS tissue samples. The CpG states were then propagated stochastically with a rate (probability per time step) of becoming methylated that increases 100-fold from a background of 10^−4^ to 10^−2^ when the mean level of methylation on the island crosses a threshold of *β* = 0.2.

Without a mathematical exploration of this Markov model, but straightforward in silico experimentation with the baseline distribution of methylation rates (specifically, a gamma distribution with mean 10^−4^ and variance 4 × 10^−8^) and threshold value, our simulations show that this simple model generates methylation density trajectories that typically bifurcate and strikingly resemble the observed drift signatures in our samples. Figure 5b shows a typical density trajectory for a region of 50 CpGs, an arrayed population of 1000 cells, every 100 time steps, for a total duration of 3000 time steps. Although our model differs in functional form from the model described in Sontag et al., it shares important features, including a suppression of de novo methylation at low levels and a nonlinear acceleration as the ambient (regional) level of methylation increases. In contrast, models that do not include this ambient methylation feedback on the local (site-specific) rate of methylation do not, in general, lead to bifurcations in the main (initial) mode of the evolving drift pattern, but still exhibit a weak bimodality as shown in Additional file 1: Figure S2. Additional file 1: Figure S3 further illustrates the stochastic nonlinear behavior of our model via simulated time course trajectories of the mean methylation levels for 10 CpG islands that share an identical drift rate distribution across their CpGs.

## Discussion

Here we take a closer look at how differential epigenetic drift is organized in BE-associated genomes, and its scope and association with gene expression, motivating further investigation of its role in neoplastic progression in BE. To do so, we first surveyed the array-based DNA methylome for significant correlations with the mean drift measured by 67 drift CpGs previously identified by our group to estimate BE dwell time, i.e., the time a patient has lived with BE [12, 13]. Following this study, we targeted CpGs that are hypomethylated in NS tissue but are subject to differential drift in BE tissue caused by accelerated age-related de novo methylation. While NS tissue may not be the tissue of origin for BE, the similarity of methylation levels at drift-associated CpGs between NS and other normal tissues, such as fundus (see Additional file 1: Figure S1), justifies the use of NS as a normal reference tissue to identify differential drift in BE. Our previous study did not reveal the full extent of this differential drift due to highly restrictive pre-filtering.

Our genome-wide “drift survey” revealed that, at the island level, > 24% of CpG islands undergo methylomic drift and are predominantly promoter-associated (i.e., overlap transcription start sites (TSS)). To investigate whether epigenetic drift occurs on isolated CpG sites or is a nonlocal phenomenon at the CpG island level, we evaluated correlations of methylation between pairs of CpGs (across BE samples) using all island-associated CpG probes (available on the HM450 platform) as a function of genomic distance between the probes (Fig. 4). Our results confirm the prevailing view that CpG islands essentially exert epigenetic control by their collective methylation state rather than through specific CpG sites [25, 26]. Importantly, we found evidence that drift does not evolve uniformly in BE and EAC but appears to be governed by a nonlinear, threshold-like stochastic methylation process which depends nonlocally on the methylation status of other island CpGs. Simulations using a stochastic model, which reflects these dynamics at the island level, show characteristic transitions from unimodal to bimodal drift similar to what we observe in our data. Although other models may provide similar fits to the observed drift distributions, this model has its origins in earlier work aimed at understanding the stable, somatic inheritance of methylation imprints [8] and predicts epigenetic drift as a series of sporadic de novo methylation events at the island level. Our nonlinear feedback model for methylomic drift suggests that the various drift distributions we see in our tissue samples may simply be attributed to tissue age itself (i.e., at what points in time the tissue samples were obtained during the dynamic process of methylomic evolution). Furthermore, analyses of consecutive biopsies in the same patient separated by several years further confirmed that epigenetic drift, as defined in this study, involves the sporadic departure from normal (hypomethylated) levels to higher levels as the tissue ages. Taken together, these findings suggest that epigenetic drift in BE advances non-uniformly by departing from unimodal (low-drift) distributions of methylation and gradually bifurcating into bimodal distributions over time. Similar unimodal and bimodal methylation distributions are observed in EAC samples although the bimodality appears more pronounced in EAC.

To investigate potential functional consequences of epigenetic drift, we compared gene expression in BE and EAC samples showing no (or low) drift to gene expression in samples that show definite drift *β* > 0.2. This comparison revealed statistically significant differences in gene expression between the two sample groups that are predominantly repressive involving several checkpoint and tumor suppressor-like genes, in particular *CHFR* (checkpoint with forkhead and ring finger domains), a mitotic stress checkpoint gene that has been observed to undergo promoter-associated hypermethylation in colon, gastric, and esophageal cancers and is associated with chromosomal instability [27, 28]. Submitting the 200 differentially repressed genes in the TCGA EAC samples to a statistical over-representation test (Additional file 2: Table S2) further revealed an unexpected high number of *KRAB* domain zinc finger genes (greater than sixfold enrichment using DAVID) that are subject to epigenetic drift and transcriptional repression possibly compromising their *KAP1*(*TRIM28*)-mediated repressive function. This finding is intriguing because *KRAB* domain ZNF also target endogenous retroviruses and transposable elements.

Finally, comparison of island-level drift with gene expression in NS and BE tissue samples from the Krause study [16] revealed that the majority of genomic loci undergoing epigenetic drift in BE are transcriptionally silent, consistent with the notion of neutral (clock-like) drift. However, the majority of differentially expressed genes associated with CpG islands that exhibit advanced drift are repressed in EAC when methylation levels increase beyond a threshold of approximately 20%. These findings support the hypothesis that neoplasia, such as dysplastic BE and EAC, may develop in response to epigenetically driven selective pressure exerted on gene expression as methylation levels (on CpG islands associated with gene promoters) advance via random drift beyond a critical, repressive threshold.

## Conclusions

Our results are consistent with the hypothesis that epigenetic drift heralds the onset of (epi)genomic instability via bifurcations (as seen in Fig. 5) that associate with the transcriptional repression of important regulatory genes [29–32]. Thus, under this hypothesis, epigenetic drift not only defines tissue aging (i.e., provides a molecular clock) but also “throttles” the expression and function of developmental genes forcing transitions in tissue characteristics that better cope with the erosive and damaging milieu in BE. Further studies of whether changes in methylomic drift simply reflect transcriptional changes during neoplastic progression or induce such changes are therefore of critical importance to better understand mechanisms that drive age-related cancer evolution.

## Methods

### Tissue samples

Formalin fixed paraffin-embedded (FFPE) tissue slides and cores were obtained from Case Western Reserve University/University Hospitals of Cleveland (Cleveland, OH) and the Cleveland Clinic (CC) following protocols approved by the Institutional Review Board of each institution. For the cross-sectional analysis, we used HM450 methylation array data from 52 NS, 64 nondysplastic BE, and 24 EAC samples through the Barrett’s Esophagus Translational Research Network (BETRNet) [33]. For the longitudinal drift analysis, we utilized 33 additional tissue samples from two studies with 10 patients each (CC and CW). Each patient had two biopsies separated by at least 3–4 years (40 samples total). Of these, seven samples were included in the cross-sectional analysis. See Additional file 2: Table S4 for relevant clinical information on the patient samples used in this study.

#### Sample pre-processing

Tissue sample preparation and DNA extraction were performed as described previously [34]. The quality of DNA extracted from FFPE samples were determined with Illumina HD FFPE QC assay (Illumina, San Diego, USA) following the manufactures’ instructions. Two hundred fifty nanograms of DNA samples that passed the QC assay were bisulfite converted using the EZ DNA Methylation Kit (Zymo Research, Irvine, USA). DNA restoration was performed using the Illumina HD FFPE Restoration Kit (Illumina, San Diego, USA) according to the manufacturer’s instructions. Intermediate DNA purifications were performed using the Zymo DNA Clean and Concentrator-5 Kit (Zymo Research, Irvine, USA). The BETRNet DNA samples were run on Illumina HumanMethylation450 BeadChip (HM450) arrays following the manufacturer’s instructions (Illumina Inc.) at the Fred Hutch Genomics Core facility. Data were then accessed as raw two-color channel intensities in *idat* format and pre-processed. Arrays were normalized using two functions implemented in the *minfi* (v1.18.6) R module, including an initial background intensity correction identical to the correction implemented in Illumina’s Genome Studio software, followed by subset quantile within-array normalization (SWAN) to harmonize data across assay design types [35, 36]. Probes showing mean detection *p* value > 0.05 were filtered out. Furthermore, we checked for the presence of previously identified cross-reactive CpGs in our drift CpG sets. Our drift CpG sets are uniformly under-enriched for cross-reactive probes, and we found that the presence of cross-reactive probes did not affect the integrity of our findings.

#### Methylation and gene expression datasets

Matched methylation (HM450 platform) and gene expression (Illumina HumanHT-12V4.0 expression BeadChip platform) data collected for 4 BE and 47 EAC, and 17 normal esophagus tissue samples published by [16] were accessed via the Gene Expression Omnibus (GEO) online repository (Series GSE72874). Methylation and expression BeadChip array data were obtained as normalized and filtered intensity counts or *β* values and prepared as described in [16].

Additional validation data, including HM450 array methylation and Illumina HiSeq 2000 RNA Sequencing data from the Version 2 analysis pipeline, were obtained for samples provided by the Cancer Genome Atlas (TCGA) via the NCI Genomic Data Commons [37] and the Firehose resource hosted by the Broad Institute (http://gdac.broadinstitute.org/). HM450 array data were obtained as raw two-color channel intensity readings, which were subjected to the same pre-processing pipeline as the BETRNet cohort data, described above. RNAseq expression sequencing data was obtained as level 3 RNAseq by expectation maximization (RSEM)-normalized and pre-processed intensity counts [38]. A nonparametric Mann-Whitney-Wilcoxon (MWW) *U* test was applied to gene-specific count data to detect differential gene expression between low methylation samples (*β* < 0.2) and advanced methylation samples (*β* ≥ 0.2).

#### Quantification of drift

The methylation state of a CpG dinucleotide on a specific chromosome is essentially a binary variable; the cytosine is either methylated or unmethylated. However, DNA methylation arrays (such as the Illumina HM450 beadchip) provide only aggregate measurement across thousands of cellular epigenomes in a given tissue sample and therefore can only provide population fractions (i.e., *β* values) of methylated probes expressed as the ratio *β* = *M*/(*M* + *U*), with *M* and *U* representing the number of methylated and unmethylated probes in the sample, respectively.

**Fig. 7:**
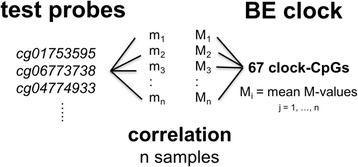
See “Methods”

Genome-wide differential epigenetic drift in BE was quantified by scanning over 146,000 hypomethylated CpG probes (*β* < 0.25 in NS tissue) on the HM450 platform for significant correlations with the mean methylation levels of 67 CpGs previously identified to drift differentially between BE and matched NS tissue samples from 30 BE patients [12]. Note, the differences in mean *M* values (defined as logit_2_(*β* value)) of the 67 drift CpGs reflect patient-specific differences in individual BE tissue dwell times as described in [12]. Figure 7 illustrates the two-step method used to identify CpG probes that were significantly correlated with this BE tissue clock: (1) we computed the mean *M* value drift over the 67 BE clock probes for each of the 64 cross-sectional BE samples and (2) for each CpG in the hypomethylated test set (146,029 CpG probes), we obtained the Pearson correlation and *p* value using the *cor.test* R-function. Only CpG probes that were significantly (*q* < 0.01) and positively (*r* > 0.5) correlated with the BE tissue clock were retained and formed the set of 18,013 island and non-island-based drift CpGs used in this study.

#### Statistical analysis and visualization

All data pre-processing and the majority of statistical testing was performed in R programming language with base R graphics and analysis functions (v3.3.0). The *minfi* (v1.18.6) and GEOquery (v2.38.4) Bioconductor modules were used to access, pre-process, normalize, and analyze both methylation and gene expression array data, respectively [35, 39, 40].

#### Data access

Data prepared for this study are available online at the GEO website (Series Number: GSE104707). Scripts for study analyses and visualizations are available at *https://github.com/gluebeck/Scope-of-methylomic-drift-in-BE*.

## Additional files

Additional file 1: Figure S1. Boxplot of normal squamous (NS) methylation fine-structure (represented using *M* values) for five representative, consecutively positioned CpGs at the *MGMT* (O-6-methylguanine-DNA methyltransferase) gene which overlaps a CpG-rich island at chr10:131264948-131265710. Mean methylation fractions (*n* = 52) range from 1% (lowest) to 19% (highest) for the five promoter-associated CpGs shown. Superimposed are the *M* values of 12 normal tissue samples collected in fundus (red). Nearly identical methylation patterns were observed in normal colon samples (not shown). Figure S2. Simulated methylation densities (arbitrary time scale) using a linear drift model without ambient methylation feedback on the rate of site-specific methylation. Figure S3. Simulated trajectories of mean methylation levels for 10 islands with 50 CpGs each under the nonlinear (threshold) model described in the main text. As methylation levels approach the threshold of *β* = 0.2, rapid stochastic transitions occur followed by accelerated drift. Figure S4 Karyograph showing locations of methylomic drift across 64 BE samples for all 22 autosomes. Figure S5. The same as Fig. 5a, but for 87 EAC from TCGA. (DOCX 863 kb)

Additional file 2: Table S1. List of genes with differential expression among low- and high-drift samples in TCGA (*n* = 87) using a *β* value threshold of 0.2 to delineate the two groups. Two hundred genes were significantly underexpressed in the advanced drift group, 15 genes (not shown) were significantly overexpressed (*q* < 0.01, Mann-Whitney-Wilcoxon two-sided test). Highlighted genes were also found to be independently and significantly underexpressed in the combined set of 47 EAC and 4 BE samples for which both gene expression and DNA methylation data were available used by Krause et al. (Carcinogenesis 37(4), 2016). **Table S2.** DAVID enrichment analysis (by protein class) of the 200 repressed genes listed in Additional file 2: Table 1. Highlighted protein classes are significantly enriched. **Table S3** CpG dinucleotide methylation transition rates for 20 patients with longitudinally collected BE biopsy samples separated by at least 3–4 years, including 10 patients from BETRNet/CC and 10 patients from BETRNet/CW. A threshold of *β* = 0.2 was used to classify CpG methylation as low (1) or high (2). The first two columns provide patient ages at biopsy, third is a patient label, columns 4–7 represent CpG fractions that begin and end at low methylation (n_11_), transition from high to low (n_21_), transition from low to high (n_12_), and remain high (n_22_). Conditional transition fractions are in columns 8–11, and annual increasing and decreasing methylation rates are in columns 12–13. **Table S4.** Patient ID (encoded), project (BETRNet/MEMO), tissue type (normal squamous (NS), Barrett’s esophagus (BE), esophageal adenocarcinoma (EAC)), sex, age at biopsy, and patient diagnosis (Dx) at the time of biopsy. (DOCX 723 kb)

## References

[1] Campisi J (2013). Aging, cellular senescence, and cancer. Annu Rev Physiol.

[2] Hannum G, Guinney J, Zhao L, Zhang L, Hughes G, Sadda S, et al. Genome-wide methylation profiles reveal quantitative views of human aging rates. Mol Cell 2013;49(2):359-67. Epub 2012/11/28. doi: 10.1016/j.molcel.2012.10.016. PubMed PMID: 23177740. 10.1016/j.molcel.2012.10.016 PMC3780611 23177740

[3] Horvath S. DNA methylation age of human tissues and cell types. Genome biology. 2013;14(10). doi: Artn R115 Doi 10.1186/Gb-2013-14-10-R115. PubMed PMID: ISI:000329387500008. 10.1186/gb-2013-14-10-r115 PMC4015143 24138928

[4] Alisch RS, Barwick BG, Chopra P, Myrick LK, Satten GA, Conneely KN (2012). Age-associated DNA methylation in pediatric populations. Genome Res.

[5] Ahuja N, Li Q, Mohan AL, Baylin SB, Issa JPJ (1998). Aging and DNA methylation in colorectal mucose and cancer. Cancer Research.

[6] Toyota M, Sasaki Y, Satoh A, Ogi K, Kikuchi T, Suzuki H (2003). Epigenetic inactivation of CHFR in human tumors. Proc Natl Acad Sci USA.

[7] Issa JP, Ahuja N, Toyota M, Bronner MP, Brentnall TA. Accelerated age-related CpG island methylation in ulcerative colitis. Cancer Research 2001;61(9):3573-7. Epub 2001/04/28. PubMed PMID: 11325821. 11325821

[8] Sontag LB, Lorincz MC, Georg Luebeck E. Dynamics, stability and inheritance of somatic DNA methylation imprints. J Theor Biol. 2006;242(4):890-9. Epub 2006/06/30. doi: 10.1016/j.jtbi.2006.05.012. PubMed PMID: 16806276. 10.1016/j.jtbi.2006.05.012 16806276

[9] Shibata D (2011). Mutation and epigenetic molecular clocks in cancer. Carcinogenesis.

[10] Issa JP. Aging and epigenetic drift: a vicious cycle. J Clin Invest 2014;124(1):24-9. Epub 2014/01/03. doi: 10.1172/JCI69735. PubMed PMID: 24382386; PubMed Central PMCID: PMC3871228. 10.1172/JCI69735 PMC3871228 24382386

[11] Teschendorff AE, West J, Beck S. Age-associated epigenetic drift: implications, and a case of epigenetic thrift?. Hum Mol Genet. 2013;22(R1):R7-R15. Epub 2013/08/07. doi: 10.1093/hmg/ddt375. PubMed PMID: 23918660; PubMed Central PMCID: PMCPMC3782071. 10.1093/hmg/ddt375 PMC3782071 23918660

[12] Curtius K, Wong CJ, Hazelton WD, Kaz AM, Chak A, Willis JE (2016). A molecular clock infers heterogeneous tissue age among patients with Barrett's esophagus. PLoS Comput Biol.

[13] Hazelton WD, Curtius K, Inadomi JM, Vaughan TL, Meza R, Rubenstein JH, et al. The role of gastroesophageal reflux and other factors during progression to esophageal adenocarcinoma. Cancer Epidemiol Biomarkers Prev. 2015. doi: 10.1158/1055-9965.EPI-15-0323-T. PubMed PMID: 25931440. 10.1158/1055-9965.EPI-15-0323-T PMC4491004 25931440

[14] Kong CY, Kroep S, Curtius K, Hazelton WD, Jeon J, Meza R, et al. Exploring the recent trend in esophageal adenocarcinoma incidence and mortality using comparative simulation modeling. Cancer Epidemiol biomarkers Prev 2014;23(6):997-1006. Epub 2014/04/03. doi: 10.1158/1055-9965.EPI-13-1233. PubMed PMID: 24692500; PubMed Central PMCID: PMC4048738. 10.1158/1055-9965.EPI-13-1233 PMC4048738 24692500

[15] Weinstein JN, Collisson EA, Mills GB, Shaw KRM, Ozenberger BA, Ellrott K (2013). The Cancer Genome Atlas Pan-Cancer analysis project. Nat Genet.

[16] Krause L, Nones K, Loffler KA, Nancarrow D, Oey H, Tang YH (2016). Identification of the CIMP-like subtype and aberrant methylation of members of the chromosomal segregation and spindle assembly pathways in esophageal adenocarcinoma. Carcinogenesis.

[17] Sandoval J, Heyn H, Moran S, Serra-Musach J, Pujana MA, Bibikova M (2011). Validation of a DNA methylation microarray for 450,000 CpG sites in the human genome. Epigenetics.

[18] Vilkaitis G, Suetake I, Klimasauskas S, Tajima S. Processive methylation of hemimethylated CpG sites by mouse Dnmt1 DNA methyltransferase. The Journal of biological chemistry. 2005;280(1):64-72. Epub 2004/10/29. doi: 10.1074/jbc.M411126200. PubMed PMID: 15509558. 10.1074/jbc.M411126200 15509558

[19] Appanah R, Dickerson DR, Goyal P, Groudine M, Lorincz MC. An unmethylated 3′ promoter-proximal region is required for efficient transcription initiation. PLoS Genet. 2007;3(2):241-53. ARTN e27 doi: 10.1371/journal.pgen.0030027. PubMed PMID: WOS:000244711700009. 10.1371/journal.pgen.0030027 PMC1797817 17305432

[20] Sanbhnani S, Yeong FM. CHFR: a key checkpoint component implicated in a wide range of cancers. . 2012;69(10):1669-1687. doi: 10.1007/s00018-011-0892-2. PubMed PMID: WOS:000303509800012. 10.1007/s00018-011-0892-2 PMC11114665 22159584

[21] Song AQ, Ye JL, Zhang KP, Yu HS, Gao YH, Wang HF (2015). Aberrant expression of the CHFR prophase checkpoint gene in human B-cell non-Hodgkin lymphoma. Leuk Res.

[22] Cancer Genome Atlas Research N, Analysis Working Group: Asan U, Agency BCC, Brigham, Women's H, Broad I (2017). Integrated genomic characterization of oesophageal carcinoma. Nature.

[23] Groner AC, Meylan S, Ciuffi A, Zangger N, Ambrosini G, Denervaud N, et al. KRAB-zinc finger proteins and KAP1 can mediate long-range transcriptional repression through heterochromatin spreading. PLoS genetics. 2010;6(3). ARTN e1000869 doi: 10.1371/journal.pgen.1000869. PubMed PMID: WOS:000276311400023. 10.1371/journal.pgen.1000869 PMC2832679 20221260

[24] Jacobs FMJ, Greenberg D, Nguyen N, Haeussler M, Ewing AD, Katzman S (2014). An evolutionary arms race between KRAB zinc-finger genes ZNF91/93 and SVA/L1 retrotransposons. Nature.

[25] Jaenisch R, Bird A (2003). Epigenetic regulation of gene expression: how the genome integrates intrinsic and environmental signals. Nature genetics.

[26] Suzuki MM, Bird A (2008). DNA methylation landscapes: provocative insights from epigenomics. Nat Rev Genet.

[27] Honda T, Tamura G, Waki T, Kawata S, Nishizuka S, Motoyama T (2004). Promoter hypermethylation of the Chfr gene in neoplastic and non-neoplastic gastric epithelia. Br J Cancer.

[28] Rashid A, Issa JPJ (2004). CpG island methylation in gastroenterologic neoplasia: a maturing field. Gastroenterology.

[29] Galhotra S, Bhattacharjee JK, Agarwalla BK. Turing-Hopf instabilities through a combination of diffusion, advection, and finite size effects. Journal of Chemical Physics. 2014;140(2). Artn 024501 doi: 10.1063/1.4859259. PubMed PMID: WOS:000329925200033. 10.1063/1.4859259 24437890

[30] Mothersill C, Seymour C (2003). Radiation-induced bystander effects, carcinogenesis and models. Oncogene.

[31] Saha AK, Tapaswi PK (1992). A stochastic reaction-diffusion model of the epigenetic system - study of localized fluctuations. Cybernetica.

[32] Quail T, Shrier A, Glass L (2015). Predicting the onset of period-doubling bifurcations in noisy cardiac systems. Proc Natl Acad Sci U S A.

[33] Abrams JA, Appelman HD, Beer DG, Berry LD, Chak A, Falk GW, et al. Barrett's Esophagus Translational Research Network (BETRNet): the pivotal role of multi-institutional collaboration in esophageal adenocarcinoma research. Gastroenterology 2014;146(7):1586-90. Epub 2014/04/29. doi: 10.1053/j.gastro.2014.04.014. PubMed PMID: 24768332. 10.1053/j.gastro.2014.04.014 PMC4224108 24768332

[34] Luo Y, Wong CJ, Kaz AM, Dzieciatkowski S, Carter KT, Morris SM, et al. Differences in DNA methylation signatures reveal multiple pathways of progression from adenoma to volorectal vancer. Gastroenterology. 2014. Epub 2014/05/06; 10.1053/j.gastro.2014.04.039. PubMed PMID: 24793120 10.1053/j.gastro.2014.04.039 PMC4107146 24793120

[35] Aryee MJ, Jaffe AE, Corrada-Bravo H, Ladd-Acosta C, Feinberg AP, Hansen KD (2014). Minfi: a flexible and comprehensive bioconductor package for the analysis of Infinium DNA methylation microarrays. Bioinformatics.

[36] Maksimovic J, Gordon L, Oshlack A. SWAN: subset-quantile within array normalization for illumina infinium HumanMethylation450 BeadChips. Genome biology. 2012;13(6). doi: Artn R44 Doi 10.1186/Gb-2012-13-6-R44. PubMed PMID: ISI:000308546300004. 10.1186/gb-2012-13-6-r44 PMC3446316 22703947

[37] Grossman RL, Heath AP, Ferretti V, Varmus HE, Lowy DR, Kibbe WA (2016). Toward a shared vision for cancer genomic data. N Engl J Med.

[38] Li B, Dewey CN. RSEM: accurate transcript quantification from RNA-Seq data with or without a reference genome. BMC bioinformatics. 2011;12. Artn 323 doi: 10.1186/1471-2105-12-323. PubMed PMID: WOS:000294361700001. 10.1186/1471-2105-12-323 PMC3163565 21816040

[39] R Core Team (2013). R: a language and environment for statistical computing.

[40] Sean D, Meltzer PS (2007). GEOquery: a bridge between the gene expression omnibus (GEO) and BioConductor. Bioinformatics.

