# Supplementary material for: Identification of a key role of widespread epigenetic drift in Barrett’s esophagus and esophageal adenocarcinoma
Source: Clin Epigenetics. 2017 Oct 16;9:113. doi: 10.1186/s13148-017-0409-4 (PMC5644061; doi:10.1186/s13148-017-0409-4)
Supplement: Supplementary file 1 — Boxplot of normal squamous (NS) methylation fine-structure (represented using M values) for five representative, consecutively positioned CpGs at the MGMT (O-6-methylguanine-DNA methyltransferase) gene which overlaps a CpG-rich island at chr10:131264948-131265710. Mean methylation fractions (n = 52) range from 1% (lowest) to 19% (highest) for the five promoter-associated CpGs shown. Superimposed are the M values of 12 normal tissue samples collected in fundus (red). Nearly identical methylation patterns were observed in normal colon samples (not shown). Figure S2. Simulated methylation densities (arbitrary time scale) using a linear drift model without ambient methylation feedback on the rate of site-specific methylation. Figure S3. Simulated trajectories of mean methylation levels for 10 islands with 50 CpGs each under the nonlinear (threshold) model described in the main text. As methylation levels approach the threshold of β = 0.2, rapid stochastic transitions occur followed by accelerated drift. Figure S4 Karyograph showing locations of methylomic drift across 64 BE samples for all 22 autosomes. Figure S5. The same as Fig. 5a, but for 87 EAC from TCGA. (DOCX 863 kb) [file 13148_2017_409_MOESM1_ESM.docx]

Additional File 1: Figure S1

Additional File 1: Figure S2

Additional File 1: Figure S3

Additional File 1: Figure S4


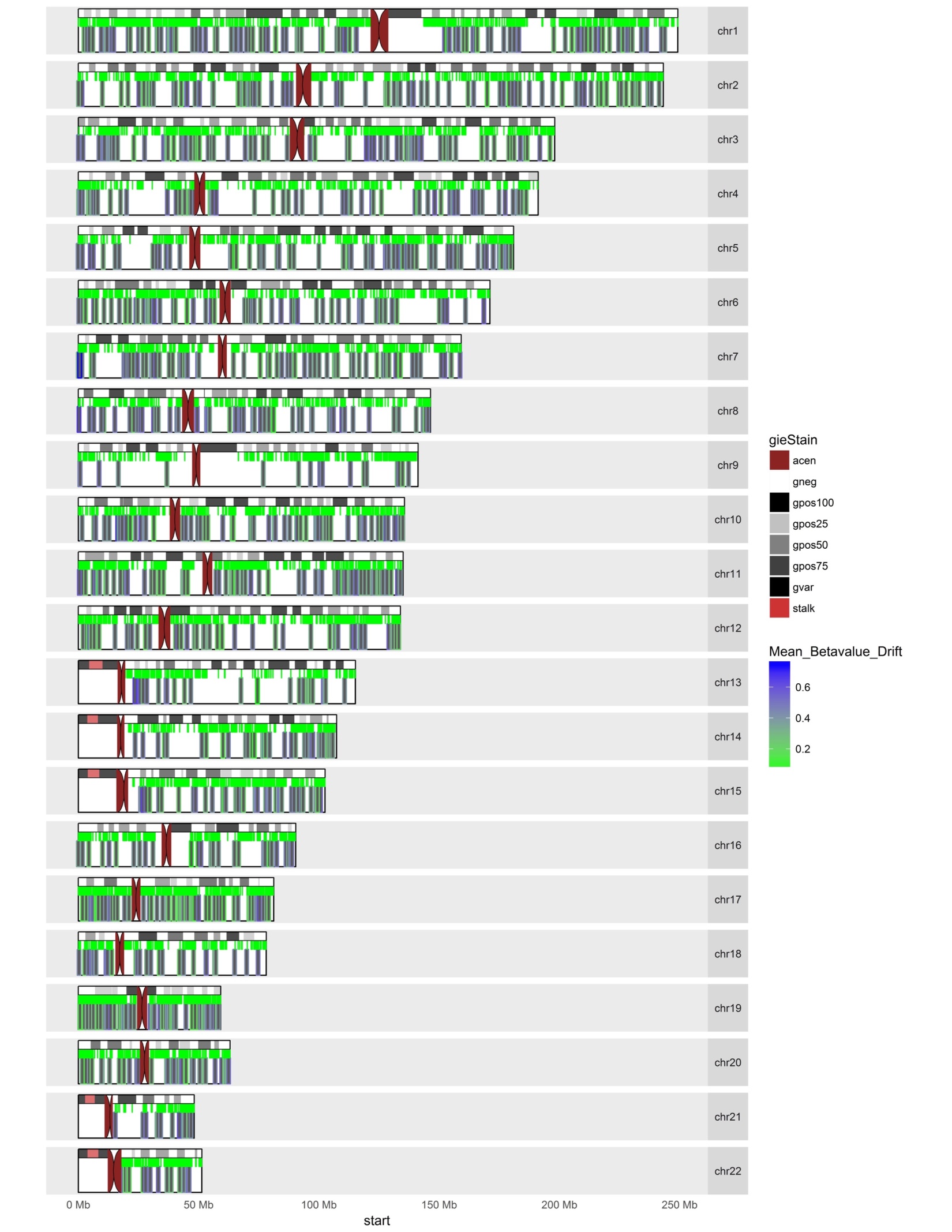


Additional File 1: Figure S5
