# Supplementary material for: Identification of a key role of widespread epigenetic drift in Barrett’s esophagus and esophageal adenocarcinoma
Source: Clin Epigenetics. 2017 Oct 16;9:113. doi: 10.1186/s13148-017-0409-4 (PMC5644061; doi:10.1186/s13148-017-0409-4)
Supplement: Supplementary file 2 — List of genes with differential expression among low- and high-drift samples in TCGA (n = 87) using a β value threshold of 0.2 to delineate the two groups. Two hundred genes were significantly underexpressed in the advanced drift group, 15 genes (not shown) were significantly overexpressed (q < 0.01, Mann-Whitney-Wilcoxon two-sided test). Highlighted genes were also found to be independently and significantly underexpressed in the combined set of 47 EAC and 4 BE samples for which both gene expression and DNA methylation data were available used by Krause et al. (Carcinogenesis 37(4), 2016). Table S2. DAVID enrichment analysis (by protein class) of the 200 repressed genes listed in Additional file 2: Table 1. Highlighted protein classes are significantly enriched. Table S3 CpG dinucleotide methylation transition rates for 20 patients with longitudinally collected BE biopsy samples separated by at least 3–4 years, including 10 patients from BETRNet/CC and 10 patients from BETRNet/CW. A threshold of β = 0.2 was used to classify CpG methylation as low (1) or high (2). The first two columns provide patient ages at biopsy, third is a patient label, columns 4–7 represent CpG fractions that begin and end at low methylation (n11), transition from high to low (n21), transition from low to high (n12), and remain high (n22). Conditional transition fractions are in columns 8–11, and annual increasing and decreasing methylation rates are in columns 12–13. Table S4. Patient ID (encoded), project (BETRNet/MEMO), tissue type (normal squamous (NS), Barrett’s esophagus (BE), esophageal adenocarcinoma (EAC)), sex, age at biopsy, and patient diagnosis (Dx) at the time of biopsy. (DOCX 723 kb) [file 13148_2017_409_MOESM2_ESM.docx]

Additional File 2: Table S1

Additional File 2: Table S2

Additional File 2: Table S3


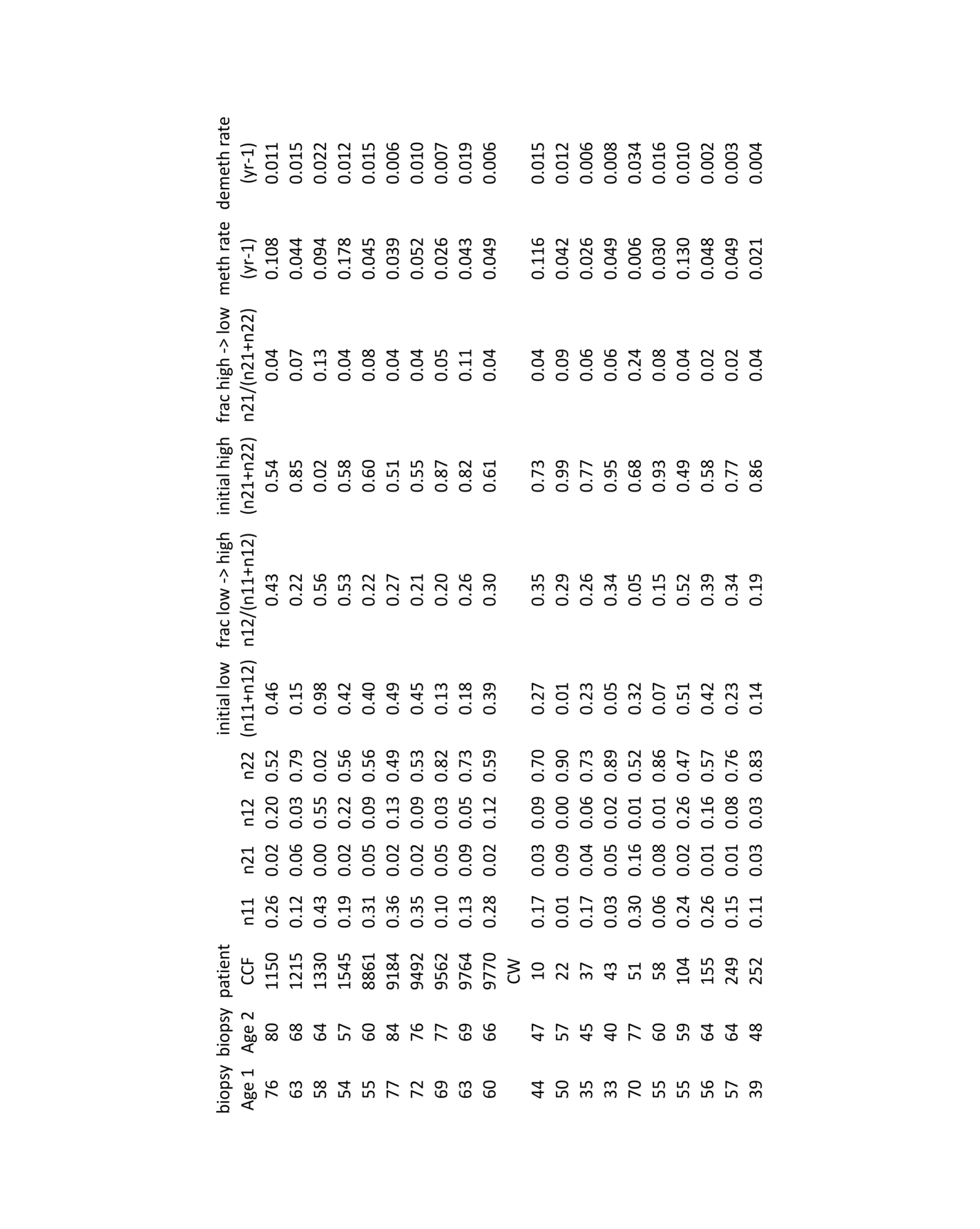


Additional File 2: Table S4 – patient/sample information

Normal Squamous (NS) samples (n=52)

| ID | project | tissue | sex | age at biopsy | Dx |
| --- | --- | --- | --- | --- | --- |
| 1 | BETRNet | SQ (3x) | Male | 64 | EAC |
| 3 | BETRNet | SQ (4x) | Male | 52 | EAC |
| NA | BETRNet | SQ | NA | NA | NA |
| 7 | BETRNet | SQ | Male | 67 | LGD |
| 23 | BETRNet | SQ | Male | 88 | EAC |
| 22 | BETRNet | SQ | Male | 71 | EAC |
| 27 | BETRNet | SQ | Female | 70 | NA |
| 28 | BETRNet | SQ | Male | 72 | EAC |
| 50 | BETRNet | SQ | Female | 43 | BE |
| 37 | BETRNet | SQ | Male | 77 | EAC |
| 35 | BETRNet | SQ | Male | 55 | HGD |
| 34 | BETRNet | SQ | Male | 68 | EAC |
| AA-1138-01-0001 | BETRNet | SQ | Male | 21 | BE |
| AA-1424-01-0001 | BETRNet | SQ | Male | 41 | BE |
| AA-1048-01-0001 | BETRNet | SQ | Male | 48 | BE |
| AA-0053-01-0001 | BETRNet | SQ | Male | 47 | BE |
| AA-0773-01-0001 | BETRNet | SQ | Female | 51 | BE |
| AA-1350-01-0001 | BETRNet | SQ | Male | 50 | BE |
| AA-1553-01-0001 | BETRNet | SQ | Female | 55 | BE |
| AA-1344-01-0001 | BETRNet | SQ | Male | 55 | BE |
| AA-1257-01-0001 | BETRNet | SQ | Male | 55 | BE |
| BB-0278-01-0001 | BETRNet | SQ | Male | 59 | BE |
| AA-1477-01-0001 | BETRNet | SQ | Male | 63 | BE |
| BB-0307-01-0001 | BETRNet | SQ | Male | 66 | BE |
| AA-1415-01-0001 | BETRNet | SQ | Male | 66 | BE |
| AA-1472-01-0001 | BETRNet | SQ | Male | 68 | BE |
| AA-1224-01-0001 | BETRNet | SQ | Male | 70 | BE |
| AA-0593-01-0001 | BETRNet | SQ | Male | 67 | BE |
| AA-1470-01-0001 | BETRNet | SQ | Male | 78 | BE |
| AA-1058-01-0001 | BETRNet | SQ | Male | 80 | BE |
| AA-0222-01-0001 | BETRNet | SQ | Male | 77 | BE |
| AA-1235-01-0001 | BETRNet | SQ | Female | 83 | BE |
| AA-1343-01-0001 | BETRNet | SQ | Male | 84 | BE |
| AA-0846-01-0001 | BETRNet | SQ | Female | 86 | BE |
| AA-1309-01-0001 | BETRNet | SQ | Male | 88 | BE |
| AA-1322-01-0001 | BETRNet | SQ | Female | 53 | BE |
| AA-1323-01-0001 | BETRNet | SQ | Male | 65 | BE |
| AA-1308-01-0001 | BETRNet | SQ | Male | 67 | BE |
| AA-1511-01-0001 | BETRNet | SQ | Male | 71 | BE |
| AA-1060-01-0001 | BETRNet | SQ | Male | 84 | BE |
| AA-1244-01-0001 | BETRNet | SQ | Female | 50 | BE |
| AA-1449-01-0001 | BETRNet | SQ | Male | 54 | BE |
| UNC023JAP_011712 | BETRNet | SQ | Male | 54 | NA |
| UNC019KHW_081610 | BETRNet | SQ | Female | 32 | NA |
| UNC028JCB_020413 | BETRNet | SQ | Male | 62 | BE |
| UNC023JAP_071613 | BETRNet | SQ | Male | 54 | NA |
| UNC021DLW_082112 | BETRNet | SQ | Male | 62 | NA |

Barrett’s esophagus (BE) samples (n=64)

| ID | project | tissue | sex | age at biopsy | Dx |
| --- | --- | --- | --- | --- | --- |
| 60 | BETRNet | BE | Male | 40 | BE |
| 59 | BETRNet | BE | Female | 71 | BE |
| 63 | BETRNet | BE | Male | 72 | BE |
| 58 | BETRNet | BE | Female | 68 | BE |
| 55 | BETRNet | BE | Male | 69 | BE |
| 54 | BETRNet | BE | Male | 69 | BE |
| 53 | BETRNet | BE | Male | 52 | BE |
| 51 | BETRNet | BE | Male | 67 | BE |
| 50 | BETRNet | BE | Female | 43 | BE |
| 65 | BETRNet | BE | Male | 70 | BE |
| 64 | BETRNet | BE | Male | 76 | BE |
| 62 | BETRNet | BE | Female | 60 | BE |
| 29 | BETRNet | BE | Male | 46 | BE |
| 50 | BETRNet | BE | Female | 43 | BE |
| 44 | BETRNet | BE | Male | 74 | BE |
| 18 | BETRNet | BE | Male | 61 | BE |
| AA-1138-01-0001 | BETRNet | BE | Male | 21 | BE |
| AA-1424-01-0001 | BETRNet | BE | Male | 41 | BE |
| AA-1048-01-0001 | BETRNet | BE | Male | 48 | BE |
| AA-0053-01-0001 | BETRNet | BE | Male | 47 | BE |
| AA-0773-01-0001 | BETRNet | BE | Female | 51 | BE |
| AA-1350-01-0001 | BETRNet | BE | Male | 50 | BE |
| AA-1553-01-0001 | BETRNet | BE | Female | 55 | BE |
| AA-1344-01-0001 | BETRNet | BE | Male | 55 | BE |
| AA-1257-01-0001 | BETRNet | BE | Male | 55 | BE |
| BB-0278-01-0001 | BETRNet | BE | Male | 59 | BE |
| AA-1477-01-0001 | BETRNet | BE | Male | 63 | BE |
| BB-0307-01-0001 | BETRNet | BE | Male | 66 | BE |
| AA-1415-01-0001 | BETRNet | BE | Male | 66 | BE |
| AA-1472-01-0001 | BETRNet | BE | Male | 68 | BE |
| AA-1224-01-0001 | BETRNet | BE | Male | 70 | BE |
| AA-0593-01-0001 | BETRNet | BE | Male | 67 | BE |
| AA-1470-01-0001 | BETRNet | BE | Male | 78 | BE |
| AA-1058-01-0001 | BETRNet | BE | Male | 80 | BE |
| AA-0222-01-0001 | BETRNet | BE | Male | 77 | BE |
| AA-1235-01-0001 | BETRNet | BE | Female | 83 | BE |
| AA-1343-01-0001 | BETRNet | BE | Male | 84 | BE |
| AA-0846-01-0001 | BETRNet | BE | Female | 86 | BE |
| AA-1309-01-0001 | BETRNet | BE | Male | 88 | BE |
| AA-1322-01-0001 | BETRNet | BE | Female | 53 | BE |
| AA-1323-01-0001 | BETRNet | BE | Male | 65 | BE |
| AA-1308-01-0001 | BETRNet | BE | Male | 67 | BE |
| AA-1511-01-0001 | BETRNet | BE | Male | 71 | BE |
| AA-1060-01-0001 | BETRNet | BE | Male | 84 | BE |
| AA-1244-01-0001 | BETRNet | BE | Female | 50 | BE |
| AA-1449-01-0001 | BETRNet | BE | Male | 54 | BE |
| 252 | BETRNet | BE | Male | 39 | BE |
| 252 | BETRNet | BE | Male | 48 | BE |
| 155 | BETRNet | BE | Male | 56 | BE |
| 155 | BETRNet | BE | Male | 64 | BE |
| 104 | BETRNet | BE | Male | 55 | BE |
| 104 | BETRNet | BE | Male | 59 | BE |
| 104 | BETRNet | BE | Male | 59 | BE |
| 104 | BETRNet | BE | Male | 64 | BE |
| 104 | BETRNet | BE | Male | 69 | BE |
| 249 | BETRNet | BE | Male | 64 | BE |
| 249 | BETRNet | BE | Male | 57 | BE |
| 251 | BETRNet | BE | Male | 67 | BE |
| UNC028JCB_020413 | BETRNet | BE | Male | 62 | BE |
| UNC017OS_041111 | BETRNet | BE | Female | 61 | BE |
| UNC021DLW_081009 | BETRNet | BE | Male | 62 | BE |
| UNC707LG_012309 | BETRNet | BE | Male | 57 | BE |
| UNC701SEJ_073008 | BETRNet | BE | Male | 80 | BE |
| UNC702RH_042709 | BETRNet | BE | Male | 72 | BE |

Esophageal Adenocarcinoma (EAC) samples (n=24)

| ID | project | tissue | sex | age at biopsy | Dx |
| --- | --- | --- | --- | --- | --- |
| 72 | BETRNet | EAC | Male | 66 | EAC |
| 1 | BETRNet | EAC | Male | 64 | EAC |
| 3 | BETRNet | EAC | Male | 52 | EAC |
| 8 | BETRNet | EAC | Male | 56 | EAC |
| 9 | BETRNet | EAC | Male | 65 | EAC |
| 11 | BETRNet | EAC | Male | 82 | EAC |
| 12 | BETRNet | EAC | Male | 77 | EAC |
| 13 | BETRNet | EAC | Male | 79 | EAC |
| 14 | BETRNet | EAC | Male | 64 | EAC |
| 24 | BETRNet | EAC | Male | 83 | EAC |
| 15 | BETRNet | EAC | Male | 48 | EAC |
| 23 | BETRNet | EAC | Male | 88 | EAC |
| 22 | BETRNet | EAC | Male | 71 | EAC |
| 4 | BETRNet | EAC | Female | 76 | EAC |
| 5 | BETRNet | EAC | Female | 49 | EAC |
| 21 | BETRNet | EAC | Male | 79 | EAC |
| 28 | BETRNet | EAC | Male | 72 | EAC |
| 31 | BETRNet | EAC | Male | 59 | EAC |
| 32 | BETRNet | EAC | Male | 54 | EAC |
| 38 | BETRNet | EAC | Male | 70 | EAC |
| 41 | BETRNet | EAC | Male | 54 | EAC |
| 37 | BETRNet | EAC | Male | 77 | EAC |
| 34 | BETRNet | EAC | Male | 68 | EAC |
| 42 | BETRNet | EAC | Female | 67 | EAC |

Longitudinal BE patients (CC study, 10x2 samples)

| ID | project | tissue | sex | age at biopsy | Dx |
| --- | --- | --- | --- | --- | --- |
| 11508227 | MEMO | BE | Male | 80 | BE |
| 11508227 | MEMO | BE | Male | 76 | BE |
| 12156022 | MEMO | BE | Female | 63 | BE |
| 12156022 | MEMO | BE | Female | 68 | BE |
| 13301158 | MEMO | BE | Male | 58 | BE |
| 13301158 | MEMO | BE | Male | 64 | BE |
| 15454753 | MEMO | BE | Female | 54 | BE |
| 15454753 | MEMO | BE | Female | 57 | BE |
| 8861439 | MEMO | BE | Male | 55 | BE |
| 8861439 | MEMO | BE | Male | 60 | BE |
| 9184732 | MEMO | BE | Male | 77 | BE |
| 9184732 | MEMO | BE | Male | 84 | BE |
| 9492038 | MEMO | BE | Male | 72 | BE |
| 9492038 | MEMO | BE | Male | 76 | BE |
| 9562150 | MEMO | BE | Male | 69 | BE |
| 9562150 | MEMO | BE | Male | 77 | BE |
| 9764119 | MEMO | BE | Male | 63 | BE |
| 9764119 | MEMO | BE | Male | 69 | BE |
| 9770801 | MEMO | BE | Male | 60 | BE |
| 9770801 | MEMO | BE | Male | 66 | BE |

Longitudinal BE patients (CW study, 10x2 samples)

| ID | project | tissue | sex | age at biopsy | Dx |
| --- | --- | --- | --- | --- | --- |
| 252 | BETRNet | BE | Male | 39 | BE |
| 252 | BETRNet | BE | Male | 48 | BE |
| 22 | BETRNet | BE | Male | 57 | EAC |
| 22 | BETRNet | BE | Male | 50 | EAC |
| 10 | BETRNet | BE | Male | 44 | HGD |
| 10 | BETRNet | BE | Male | 47 | HGD |
| 51 | BETRNet | BE | Male | 70 | BE |
| 51 | BETRNet | BE | Male | 77 | BE |
| 155 | BETRNet | BE | Male | 56 | BE |
| 155 | BETRNet | BE | Male | 64 | BE |
| 104 | BETRNet | BE | Male | 55 | BE |
| 104 | BETRNet | BE | Male | 59 | BE |
| 58 | BETRNet | BE | Female | 55 | BE |
| 58 | BETRNet | BE | Female | 60 | BE |
| 43 | BETRNet | BE | Male | 33 | BE |
| 43 | BETRNet | BE | Male | 40 | BE |
| 37 | BETRNet | BE | Male | 35 | EAC |
| 37 | BETRNet | BE | Male | 45 | EAC |
| 249 | BETRNet | BE | Male | 64 | BE |
| 249 | BETRNet | BE | Male | 57 | BE |
